# Profiling the Virulence and Antibiotic Resistance Genes of *Cronobacter sakazakii* Strains Isolated From Powdered and Dairy Formulas by Whole-Genome Sequencing

**DOI:** 10.3389/fmicb.2021.694922

**Published:** 2021-06-30

**Authors:** Julio Parra-Flores, Ondrej Holý, Francisca Riffo, Sarah Lepuschitz, Eduard Maury-Sintjago, Alejandra Rodríguez-Fernández, Ariadnna Cruz-Córdova, Juan Xicohtencatl-Cortes, Jetsi Mancilla-Rojano, Miriam Troncoso, Guillermo Figueroa, Werner Ruppitsch, Stephen Forsythe

**Affiliations:** ^1^Department of Nutrition and Public Health, Universidad del Bío-Bío, Chillán, Chile; ^2^Department of Public Health, Palacký University Olomouc, Olomouc, Czechia; ^3^Servicio de Salud Ñuble, Chillán, Chile; ^4^Austrian Agency for Health and Food Safety, Institute for Medical Microbiology and Hygiene, Vienna, Austria; ^5^Intestinal Bacteriology Research Laboratory, Hospital Infantil de México Federico Gómez, Mexico City, Mexico; ^6^Faculty of Medicine, Biological Sciences Graduate Program, Posgrado en Ciencias Biológicas, Universidad Nacional Autónoma de México, Mexico City, Mexico; ^7^Microbiology and Probiotics Laboratory, Institute of Nutrition and Food Technology, Universidad de Chile, Santiago, Chile; ^8^foodmicrobe.com, Nottinghamshire, United Kingdom

**Keywords:** *Cronobacter sakazakii*, powdered formula, virulence, antibiotic resistance genes, CRISPR-cas

## Abstract

*Cronobacter sakazakii* is an enteropathogen that causes neonatal meningitis, septicemia, and necrotizing enterocolitis in preterm infants and newborns with a mortality rate of 15 to 80%. Powdered and dairy formulas (P-DF) have been implicated as major transmission vehicles and subsequently the presence of this pathogen in P-DF led to product recalls in Chile in 2017. The objective of this study was to use whole genome sequencing (WGS) and laboratory studies to characterize *Cronobacter* strains from the contaminated products. Seven strains were identified as *C. sakazakii*, and the remaining strain was *Franconibacter helveticus*. All *C. sakazakii* strains adhered to a neuroblastoma cell line, and 31 virulence genes were predicted by WGS. The antibiograms varied between strains. and included *mcr-9.1* and *bla*_*CSA*_ genes, conferring resistance to colistin and cephalothin, respectively. The *C. sakazakii* strains encoded I-E and I-F CRISPR-Cas systems, and carried IncFII(pECLA), Col440I, and Col(pHHAD28) plasmids. In summary, WGS enabled the identification of *C. sakazakii* strains and revealed multiple antibiotic resistance and virulence genes. These findings support the decision to recall the contaminated powdered and dairy formulas from the Chilean market in 2017.

## Introduction

On June 2, 2017, the Chilean Ministry of Health issued a national and international food safety alert as a result of the presence of *Cronobacter sakazakii* in one batch of powdered infant formula (PIF) and one batch of dairy products (powder milk). This preventive measure was adopted due to the risk of disease associated with *C. sakazakii* in vulnerable populations (Parra-Flores et al., 2018b).

Cronobacter is a genus of pathogens formerly known as Enterobacter sakazakii and is now made up of seven species: C. sakazakii, C. malonaticus, C. universalis, C. turicensis, C. muytjensii, C. dublinensis, and C. condimenti (Iversen et al., 2008; Joseph et al., 2012; Stephan et al., 2014). The most susceptible population groups are newborns younger than 12 months and the elderly (Hariri et al., 2013; Patrick et al., 2014). The clinical profile is mainly meningitis, septicemia, or necrotizing enterocolitis, with a mortality ranging from 15 to 80% (Kleiman et al., 1981; Holý and Forsythe, 2014). The disease is associated with the consumption of contaminated rehydrated PIF. The pathogen has also been isolated from dairy products, infant cereals, milk substitutes, water, food preparation surfaces, and expressed breast milk (Baumgartner et al., 2009; Parra et al., 2015; Vojkovska et al., 2016; Morato-Rodríguez et al., 2018). The source of contamination is closely associated with powdered milk (PM) manufacturing plants and the ingredients used in its manufacture (Holý and Forsythe, 2014). Internationally, the incidence of *Cronobacter* spp. in PM ranges from 3 to 30% (Ling et al., 2021). In Chile, its incidence in PIF was 9.5% in 2015, 35% in 2017, and 4.7% in 2020 (Parra et al., 2015; Parra-Flores et al., 2018b, 2020).

The genus *Cronobacter* has diversified over the course of its evolution, with some species pathogenic to humans and other species whose impact on human health is still unknown (Forsythe, 2018). *C. sakazakii* and *C. malonaticus* were reported are the species with the highest clinical significance, having been involved in cases and outbreaks reported in the literature (Forsythe, 2018; Parra-Flores et al., 2021). However, information on the diversity, pathogenicity, and virulence of *Cronobacter* species obtained from various sources is still poorly understood.

Key aspects for the prognosis and development of the disease caused by *C. sakazakii* include the presence of virulence factors which may be plasmidborne (Shi et al., 2018; Aly et al., 2019), its adherence and invasiveness in cell lines (Cruz et al., 2011; Parra-Flores et al., 2018a; Holý et al., 2021), the presence of genes such as *ompA*, *cpa*, *fliC*, *hly*, *sip*, *aut*, *plas*, and *inv* (Cruz et al., 2011; Franco et al., 2011; Aldubyan et al., 2017; Holý et al., 2019), sialic acid utilization, as well as its capsule and endotoxin production (Ogrodzki and Forsythe, 2015). Another key aspect is resistance to beta-lactam antibiotics such as cephalothin, cefotaxime, ceftazidime, and ampicillin (Flores et al., 2011; Lee et al., 2012; Fei et al., 2017; Holý et al., 2021).

Whole-genome sequencing (WGS) studies generate a high degree of information content for pathogenic strains, including an accurate understanding of the taxonomic differences between them. WGS is used as a tool to identify and genotype pathogens (MLST, CRISPR-Cas, serogroup), as well as predict antibiotic-associated and virulence genes. Thus allowing more precise epidemiological links to be established (Leopold et al., 2014). WGS analysis and genome comparison, in addition to the use of *in vivo* and *in vitro* models, provide us with more accurate information about the pathogenic potential of *C. sakazakii* (Lehner et al., 2018).

In this study, we used WGS and laboratory studies to characterize the virulence, and antibiotic resistance genes of *C. sakazakii* strains isolated from powdered infant formula and powdered milk.

## Materials and Methods

### Strains Used in the Study

Eight suspected strains of *Cronobacter* isolated in 2017 from powdered infant formula and powdered milk were used. The strains were isolated after pre-enrichment in buffered peptone water (BPW) followed by *Enterobacteriaceae* enrichment broth (BD Difco, Sparks, MD, United States), isolation on Brilliance chromogenic agar CM 1035 (Oxoid Thermo-Fisher, United Kingdom) and purification on trypticase soy agar (BD Difco, Sparks, MD, United States) (Parra-Flores et al., 2018b). The isolates were presumptive identified as *C. sakazakii* or *Cronobacter* species unknown. using polymerase chain reaction (PCR) probes by gene amplification of *16S* (Lehner et al., 2004), *ompA* (Mohan-Nair and Venkitanarayanan, 2006), *rpoB* (Stoop et al., 2009), *cgcA* (Carter et al., 2013), and *fusA* gene sequencing (Baldwin et al., 2009).

### Reidentification of *Cronobacter* Isolates

For reidentification, the eight isolates were cultured on Columbia blood agar plates (bioMérieux, Marcy-l’Étoile, France) at 37°C for 24 h. Primary species identification from single colonies was carried out by matrix-assisted laser desorption ionization - time-of-flight mass spectrometry (MALDI-TOF-MS) (Bruker, Billerica, MA, United States) and MBT Compass IVD software 4.1.60 (Bruker), described by Lepuschitz et al. (2017).

### Whole-Genome Sequencing

High quality genomic DNA from an overnight culture was obtained using the MagAttract high molecular weight (HMW) DNA kit (Qiagen, Hilden, Germany). The quantification of input DNA was performed with a Qubit 2.0 fluorometer (Thermo Fisher Scientific, Waltham, MA, United States) and the double-stranded DNA (dsDNA) BR assay kit (Thermo Fisher Scientific). WGS of *C. sakazakii* strains was performed as described (Lepuschitz et al., 2019). Default parameters were used for all software unless otherwise specified. Raw reads were quality controlled using FastQC v0.11.9. Trimmomatic v0.36 (Bolger et al., 2014) was used to remove adapter sequences and to trim the last 10 bp of each sequence and sequences with quality scores <20. Reads were assembled using SPAdes v3.11.1 (Bankevich et al., 2012). Contigs were filtered for a minimum coverage of 5× and a minimum length of 200 bp using SeqSphere + software v6.0.0 (Ridom GmbH, Würzburg, Germany).

A total of 3,678 targets were used to establish the core genome multilocus sequence typing (cgMLST) scheme using strain ATCC BAA-894 as reference. According to the determined cgMLST scheme, the genotypic relationships of isolates was visualized using a minimum spanning tree (MST) as per Lepuschitz et al. (2019). In addition, the sequences of the seven housekeeping genes (*atpD, fusA, glnS, gltB, gyrB, infB*, and *ppsA*) of the conventional multilocus sequence typing (MLST) scheme were extracted and compared with the *Cronobacter* MLST database, from which sequence types (STs) were assigned *in silico* (Forsythe et al., 2014). The strains in this study are ID 3196–3202 in the *Cronobacter* PubMLST database.

### O-Serotyping

The presence of the serotype O region-specific *gnd* and *galF* genes was determined by WGS sequence analysis with the BIGSdb tool present in the PubMLST database^1^.

### Cell Line Adhesion and Invasion Assay

The mouse neuroblastoma cell line N1E-115 (American Type Culture Collection, Manassas, VA, United States) was used for the assay. The N1E-115 cell line was cultured in Dulbecco’s modified Eagle medium (DMEM) supplemented with 4.5 g/L glucose (GIBCO, United States) and 7% fetal bovine serum (FBS) (GIBCO, United States). They were then differentiated in DMEM medium supplemented with 2% FBS and 1.25% dimethyl sulfoxide for 5 days. Cells were seeded in 24-well plates (Corning Life Sciences, United States) at 1 × 10^5^ cells/ml and infected at 100:1 multiplicity of infection with each *C. sakazakii* isolate after culture in Luria broth. Infection was carried out for 4 h at 37°C in 5% CO_2_. After incubation, the cells were washed with 1 × phosphate-buffered saline (PBS), and *C. sakazakii* was removed by the addition of 1 ml of 0.1% Triton X-100 (Amresco, OH, United States). To quantify the colony-forming units (CFU) of bacteria attached to the N1E-115 cell, various dilutions were performed in Luria broth (Cruz et al., 2011).

For the invasion assay, the preparation of the N1E-115 monolayers and the time for infection were as described for the adhesion assay. After 4 h of incubation, the infected monolayers were washed with 1 × PBS and incubated with 1 ml of DMEM plus 300 μg/ml lysozyme (Sigma-Aldrich, United States) and 100 μg/ml gentamicin (Sigma-Aldrich, United States) for 2 h at 37°C in 5% CO_2_. The cells were washed three times with 1 × PBS, separated with 1 ml of 0.1% Triton X-100, and plated on Luria-Bertani agar. Invasion frequencies were calculated as the number of bacteria that survived incubation with gentamicin and lysozyme divided by the total number of bacteria present in the absence of this antibiotic (bacterial adherence) (Cruz et al., 2011).

Both assays (adhesion, invasion) were repeated twice and performed in duplicate. The data are expressed as the means.

### Detection of Virulence Factors *in vitro*

Seven genes were detected by PCR. The genes evaluated were plasminogen activator (*cpa*), presence of hemolysin (*hly*), siderophore-interacting protein (*sip*), invasin/intimin *(inv*), flagellin (*fliC*), autotransporter (*aut*), and outer membrane protein (*ompA*). The amplified products were stained and visualized on 1.5% agarose gel with 1.0 mg/ml ethidium bromide solution using an agarose gel imaging system (Mohan-Nair and Venkitanarayanan, 2006; Cruz et al., 2011; Holý et al., 2019).

### Antibiotic Resistance Profile

The disk diffusion method was used in accordance with the recommendations of the Clinical and Laboratory Standards Institute (Clinical and Laboratory Standards Institute [CLSI], 2018). Commercial antibiotic disks consisting of ceftazidime (30 μg), cefotaxime (30 μg), amoxicillin-clavulanic acid (20/10 μg), ciprofloxacin (5 μg), cephalothin (30 μg), nalidixic acid (35 μg), gentamicin (10 μg), tetracycline (30 μg), chloramphenicol (30 μg), and ampicillin (10 μg) were used. Resistance/susceptibility profiles were characterized according to the manufacturer’s instructions. *E. coli* strain ATCC 25922 was used as quality control.

### *In silico* Detection of Virulence and Antibiotic Resistance Genes

The existence of virulence genes was confirmed by using the task template feature in SeqSphere + for WGS data and the ResFinder tool from the Center for Genomic Epidemiology (CGE)^2^. Thresholds for the target scanning procedure were set as a required identity of ≥90% with the reference sequence and an aligned reference sequence ≥99%. For antimicrobial resistance genes, the Comprehensive Antibiotic Resistance Database was used with the default “perfect” and “strict” settings for sequence analysis (Jia et al., 2017), and Task Template AMRFinderPlus 3.2.3, available in Ridom SeqSphere + 7.0 software, was used with the EXACT method at the 100% setting and with BLAST alignment of protein sequences against the AMRFinderPlus database.

### Plasmid Detection

The PlasmidFinder 2.1 and MobileElementFinder 1.0 tool were used to detect plasmids. We chose a minimum identity of 95 and 90%, respectively (see text footnote 2) (Carattoli et al., 2014; Johansson et al., 2021).

### Profiling of CRISPR-Cas Loci

The search and characterization of CRISPR arrays and their association to Cas proteins was determined with CRISPRCasFinder and CRISPRDetect (Biswas et al., 2016; Couvin et al., 2018), available from the Institut de Biologie Intégrative de la Cellule in the Université Paris-Saclay server^3^ and University of Otago^4^. The types pf CRISPR systems were determinated with the CRISPRmap program (Lange et al., 2013).

### Statistical Analysis

Statistical significance (*p* < 0.05) was determined using Student’s *t*-test for the adherence and invasion assays.

## Results and Discussion

The eight strains initially identified in 2017 as *Cronobacter* were reidentified with MALDI-TOF MS as seven strains of *Cronobacter sp.* and one strain of *Franconibacter helveticus* (Table 1). At the time of original analysis, strain CH85 *fusA* gene sequence did not correspond with any known *Cronobacter* species. However, in the original paper (Parra-Flores et al., 2018b) it was designated as ‘*Cronobacter* spp.’ based on the results of the PCR probes. Due to the later recognition of the *Franconibacter* genus, the *fusA* sequence is now designated as ‘*Franconibacter helveticus*’ and this indicates that the original PCR probes lacked genus specificity. Subsequently, from WGS data and using average nucleotide identity (ANI), ribosomal MLST, and core genome MLST, six strains were confirmed as *C. sakazakii* ST1, one as *C. sakazakii* ST83, and the remaining strain as *Franconibacter helveticus* ST345. *F. helveticus* has previously been mis-identified as *Cronobacter* as they are closely related (Stephan et al., 2014; Jackson and Forsythe, 2016). In addition, the *C. sakazakii* strains identified as ST1 had the same O:1 serotype and the same gene loci for the O-antigen flanking genes *gnd* and *galF* (*galF* 2; *gnd* 1), in contrast to the ST83 isolate, whose loci were *galF* 21 and *gnd* 65.

**TABLE 1:** Re-identification of strains by MALDI-TOF and whole genome sequencing (WGS).

**Strains**	**NGS ID^1^**	**PubMLST ID**	**Presumptive identification^2^**	**MALDI-TOF**	**WGS^3^**	**ST^4^**	**CC^5^**
CH42	510289-18	3195	*C. sakazakii*	*Cronobacter sp.*	*C. sakazakii*	1	1
CH43	510291-18	3196	*C. sakazakii*	*Cronobacter sp.*	*C. sakazakii*	1	1
CH44	510293-18	3197	*C. sakazakii*	*Cronobacter sp.*	*C. sakazakii*	1	1
CH45	510295-18	3198	*C. sakazakii*	*Cronobacter sp.*	*C. sakazakii*	1	1
CH50	510296-18	3199	*C. sakazakii*	*Cronobacter sp.*	*C. sakazakii*	83	83
CH65	510296-18	3200	*C. sakazakii*	*Cronobacter sp.*	*C. sakazakii*	1	1
CH84	510298-18	3201	*C. sakazakii*	*Cronobacter sp.*	*C. sakazakii*	1	1
CH85	510439-19	3202	*Cronobacter* spp.	*Franconibacter helveticus*	*Franconibacter helveticus*	345	−

*^1^NGS ID, next generation sequencing identification; ^2^Parra-Flores et al. (2018b); ^3^ANI, Average nucleotide identity, ribosomal MLST, and core genome MLST; ^4^ST, sequence type; ^5^CC, clonal complex.*

**FIGURE 1:**
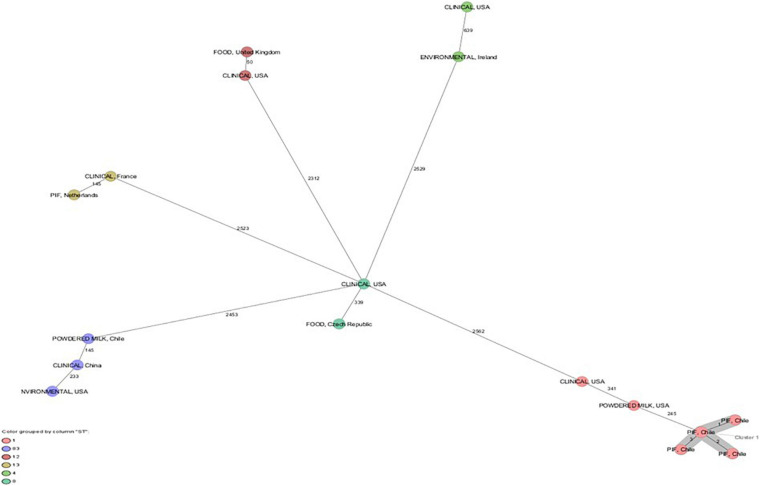
Minimum spanning tree (MST) of seven *Cronobacter sakazakii* strains from powdered infant formula and powdered milk isolated in Chile. In addition, *C. sakazakii* strains with ST1, ST4, ST8, ST12, ST13, and ST83 of clinical origin and food. Calculation of the MST was based on the defined cgMLST scheme comprising 3678 target genes from ATCC BAA-894. Isolates are represented as colored circles according to the classical MLST. Black numbers accord to the allelic difference between isolates. Isolates with closely related genotypes are marked as Cluster.

The cgMLST scheme analysis revealed a cluster of six *C. sakazakii* ST1 strains with one to three allele differences and ST83 strain with 3,043 allele difference (Figure 1). *C. sakazakii* ST1 has been most frequently isolated from commercial PIF in various countries, from processing equipment for PIF manufacturing, and from patients with fatal meningitis, septicemia, or urinary tract infections (Joseph and Forsythe, 2012; Fei et al., 2015; Holý et al., 2021; Parra-Flores et al., 2021). Lepuschitz et al. (2017), in their multicenter study of infections caused by *C. sakazakii* in Europe, studied eight isolates identified as *C. sakazakii* ST1 that included two isolates from newborns’ feces with an epidemiological link to an outbreak in Austria in 2009. Furthermore, these eight isolates showed one allelic difference and were closely related to strain ATCC BAA-894, a strain isolated from a powdered infant formula in the United States in 2001, which was associated with an outbreak involving two newborns with necrotizing enterocolitis hospitalized in the same neonatal intensive care unit. *C. sakazakii* ST83 has also been isolated from severe clinical cases, in addition to being found in PIF and the environment, so it has been suggested that *C. sakazakii* strains ST4, ST1, ST8, ST12, and ST83 are the most likely to produce disease in infants and children (Himelright et al., 2002; Sonbol et al., 2013; Fei et al., 2017; Chase et al., 2017; Forsythe, 2018).

**FIGURE 2:**
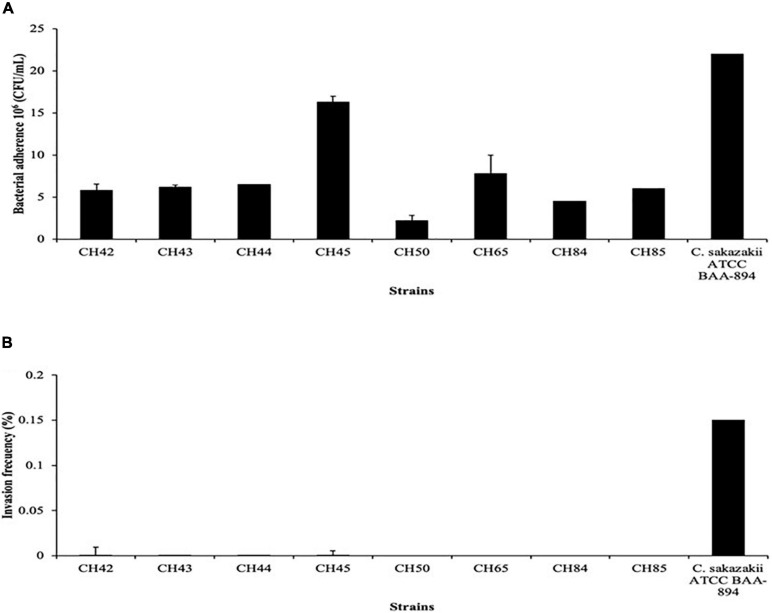
Bacterial adherence **(A)** and invasion frequency **(B)** of *Cronobacter sakazakii* strains on neuroblastoma (NT) cell line.

Several authors have studied the invasiveness and adhesion processes *in vitro* in different cell lines, such as CaCo-2, HEp-2, HBMECs, IEC-6, and N1E-115, as an initial stage of pathogenesis by *C. sakazakii* (Mange et al., 2006; Cruz et al., 2011, Holý et al., 2019). Adherence, as a first step, alters the epithelium, making it possible for the pathogen to cross the mucosa and then migrate to the bloodstream (Hunter and Bean, 2013). In our study, 100% of the strains adhered the N1E-115 cell line with ranges from 2.2 to 16.3 × 10^6^ CFU/mL. Strain CH45 (ST1) was the most adherent and strain CH50 (ST83) the least. In the invasion assay only four strains (50%) invaded with frequencies from 0.0002 to 0.00009% (Figure 2). These values are similar to those reported by Holý et al. (2019), where 100% and 66.7% of the *C. sakazakii* clinical strains evaluated adhered to and invaded the N1E-115 cell line, respectively. Recently, Holý et al. (2021), when evaluating clinical strains of *C. sakazakii* with the N1E-115 cell line, found a value of 2.2 × 10^6^ CFU/mL for adherence, which is much lower than those found in our study. However, the rates of invasion of *C. sakazakii* in our study are lower than those reported by various authors, such as Mange et al. (2006); Townsend et al. (2007), Parra-Flores et al. (2018a), and Holý et al. (2019). Recently, Veronica et al. (2020) found that N1E-115 neuroblastoma cells are highly permissive to infection by a wild-type *C. sakazakii* strain, suggesting that flagellum and outer membrane proteins are necessary to promote invasion of N1E-115 cells, but not adherence.

In regard to the presence of putative virulence factors by PCR, all *C. sakazakii* strains were positive for six genes (*hlyA*, *ompA*, *aut*, *fliC*, *sip*, and *cpa*), and all were negative for the *inv* gene (Table 2). Holý et al. (2019) found that only 76% of strains expressed the *inv* gene among strains with good levels of adherence but low invasion rates in HT-29 and N1E-115 cell lines. The *inv* gene encodes a protein mediating bacterial adhesion and basolateral and apical invasion in epithelial cells. The main function of this protein is as an invasin and was previously described by Chandrapala et al. (2014).

**TABLE 2:** Results for putative virulence genes among *Cronobacter sakazakii* strains.

**Strain**	**Putative virulence genes^a^**						
	***hlyA***	***ompA***	***Aut***	***fliC***	***Inv***	***Sip***	***Cpa***
CH42 (ST1)	+	+	+	+	−	+	+
CH43 (ST1)	+	+	+	+	−	+	+
CH44 (ST1)	+	+	+	+	−	+	+
CH45 (ST1)	+	+	+	+	−	+	+
CH50 (ST83)	+	+	+	+	−	+	+
CH65 (ST1)	+	+	+	+	−	+	+
CH84 (ST1)	+	+	+	+	−	+	+
ATCC BAA-894 (ST1)	+	+	+	+	+	+	+

*^*a*^Genes of: *hlyA*, hemolysin; *ompA*, outer membrane protein A; *au*t, autotransporter; *fliC*, flagellin; *inv*, invasin/intimin; *sip*, siderophore-interacting protein; *cpa*, plasminogen activator.*

Thirty-one virulence genes were detected *in silico*, which were grouped into flagellar proteins, outer membrane proteins (*ompA*), chemotaxis (*motB*), hemolysins (*hly*III), invasion (*lpxA*), plasminogen activator (*cpa*), colonization (*mviM*), transcriptional regulator (*sdiA*), macrophage survival, sialic acid utilization (*nanA*, *nanK*), and toxins-antitoxins (TA) (*fic*) (Table 3). These results are consistent with PCR findings, with the exception of the *inv* gene, which was not detected by PCR. However, invasion genes such as *lpx* were present in the genome, and therefore further studies are needed to evaluate the specificity of the *inv* gene.

**TABLE 3:** Putative virulence and other genes distribution among seven strains of *Cronobacter sakazakii* by whole-genome sequencing (WGS).

**Virulence gene**	**Function**	**CH42 (ST1)**	**CH43 (ST1)**	**CH44 (ST1)**	**CH45 (ST1)**	**CH50 (ST83)**	**CH65 (ST1)**	**CH84 (ST1)**	**ES15 control (ST125)**	***C. sakazakii* BAA-894 (ST1)**
*flgB*	motility	+	+	+	+	+	+	+	+	+
*flgK*	flagellar hook-associated protein 1	+	+	+	+	+	−	+	+	+
*flgL*	flagellar hook-associated protein 3	+	+	+	+	+	+	+	+	+
*flgM*	negative regulator of flagellin synthesis	+	+	+	+	+	+	+	+	+
*flgN*	flagella synthesis FlgN protein	+	+	+	+	+	+	+	+	+
*flhD*	flagellar hook-associated protein 2	+	+	+	+	+	+	+	+	+
*fliA*	flagellar operon FliA	+	+	+	+	+	+	+	+	+
*fliC*	flagellin	+	+	+	+	+	+	+	+	−
*fliD*	flagellar hook-associated protein 2	+	+	+	+	+	+	+	+	+
*fliR*	flagellar biosynthetic FliR protein	+	+	+	+	+	+	+	+	+
*fliT*	flagella FliT protein	+	+	+	+	+	+	+	+	+
*fliZ*	FliZ protein	+	+	+	+	+	+	+	+	+
*lolA*	outer membrane lipoprotein carrier protein	+	+	+	+	+	+	+	+	+
*motB*	chemotaxis MotA protein	+	+	+	+	+	+	+	+	+
*sdiA*	LuxR family transcriptional regulator	+	+	+	+	+	+	+	+	+
*slyB*	outer membrane lipoprotein SlyB	+	+	+	+	+	+	+	+	+
*tolC*	outer membrane channel protein	+	+	+	+	+	+	+	+	+
*msbA*	survival in macrophage	+	+	+	+	+	+	−	+	
*mviN*	protective immunity and colonization	+	+	+	+	+	+	+	+	+
*cpa*	plasminogen activator	+	+	+	+	+	+	+	−	+
*hha*	hemolysin expression modulating protein	+	+	+	+	+	+	−	+	+
*hly III*	hemolysin III	+	+	+	+	+	+	−	+	+
*ompA*	adhesion cell; biofilm formation	+	+	+	+	+	+	+	+	+
*ompX*	adhesion cell	+	+	+	+	+	+	+	+	+
*blc*	outer membrane lipoprotein	+	+	+	+	+	+	−	+	+
*cheR*	chemotaxis protein methyltransferase	+	+	−	+	+	+	+	+	−
*cheY*	response regulator of chemotaxis family	+	+	+	+	+	+	+	+	+
*lpxA*	epithelial cell invasion and lipid A production	+	+	+	+	+	+	+	+	+
*nanA,K,T*	utilization of exogenous sialic acid	+	+	+	+	+	+	+	+	+
*fic*	cell filamentation protein	+	+	+	+	+	+	+	+	+
*relB*	antitoxin to RelE	+	+	+	+	+	+	+	+	+

* + = presence; − = absence.*

The OmpA and OmpX proteins of *C. sakazakii* are involved in basolateral adhesion in CaCo2 and INT-407 cell lines, in addition to a possible involvement in the crossing of the blood–brain barrier by *Cronobacter* spp. (Mange et al., 2006; Kim et al., 2010). The Cpa protein is related to serum resistance and systemic spread of *C. sakazakii*. The *cpa* locus could be considered specific to *C. sakazakii* and *C. universalis* (Franco et al., 2011). However, highly virulent clinical ST8 strains of *C. sakazakii* that bear plasmid pESA3 have been found to lack the *cpa* gene, suggesting the likely presence of other virulence genes as responsible for the disease (Jang et al., 2020). Hemolysins (Hly) are outer membrane proteins or exoproteins found in various pathogens belonging to the *Enterobacteriaceae* family, such as *Escherichia coli*, *Klebsiella*, *Enterobacter*, and Gram-positive pathogens such as *Bacillus cereus*, with hemolytic capacity (Mare et al., 2020; Mazzantini et al., 2020). This *hly* gene was found when analyzing the *C. sakazakii* BAA-894 strain that was isolated from the neonatal intensive care unit outbreak in 2001 (Himelright et al., 2002).

Twelve genes associated with flagellar proteins, synthesis, operons, and flagellin (*fliC*) were found (Table 3). Their main functions are bacterial motility, adherence capacity, biofilm formation, and stimulation of proinflammatory responses through receptor TLR5 signaling (Proudy et al., 2008). Aldubyan et al. (2017), described the prevailing role of flagella in the adherence and invasion of pathogenic *fliC*-containing bacteria. However, some authors have proposed that flagellar motility in *C. sakazakii* is not necessary for biofilm formation (Ye et al., 2015), showing that self-aggregation is a biological function of the flagellum that favors decreased motility (Hoeflinger and Miller, 2017). Dingle et al. (2011) described, in *Clostridium difficile* mutant strains, the important role of the major flagellar subunits FliC and FliD in their increased adherence to Caco-2 cells and their greater virulence than wild-type. The presence of the *nanA* and *nanK* genes, encoding the ability to use of exogenous sialic acid as a carbon source, is another important virulence factor. This particular feature is considered an evolutionary adaptation of *C. sakazakii*, as this compound is found naturally in breast milk and is added supplementally to PIF due to its association with brain development as it is a major component of gangliosides (Forsythe, 2018). Sohanpal et al. (2007) demonstrated how sialic acid can modify bacterial surfaces by regulating the expression of enzymes such as sialidase and adhesins or inhibiting transcription factors of the *fimB* gene, part of the *fim* operon, which are virulence factors that mediate epithelial cell adhesion and invasion (Severi et al., 2007). Moreover, the use of sialic acid by bacterial cells has been linked to several virulence factors. The bacterial glycolipid capsule is an example of host molecular adaptation, as it helps the pathogen circumvent host immune responses. Neonatal meningitic *Escherichia coli* K1 uses sialic acid to modify its cell surface, and *Cronobacter* spp. produce capsular material when cultured in milk (Caubilla-Barron and Forsythe, 2007). However, there is no evidence of genes encoding for a sialic acid capsule in *C. sakazakii* strains.

Few reports have identified possible the presence of toxins associated with *C. sakazakii*. In our study we found the typical *fic* TA gene and the *relB* gene, which encodes the *relE* antitoxin. Toxin–antitoxin (TA) systems are small genetic elements found in plasmids, phage genomes and chromosomes of different bacterial species. Furthermore, these TA genes have a prominent role in the physiology of bacterial stress, such as in the stabilization of horizontally acquired mobile elements. They are also involved in a persistence phenotype in some species, such as *E. coli* and *Salmonella* (Deter et al., 2017; Walling and Butler, 2019). Finkelstein et al. (2019) found, in preliminary studies of *C. sakazakii* isolates, that two typical TA genes, *fic* and *hipA*, followed species-specific evolutionary lines. When expanding their focus to evaluate the presence of five TA in *C. sakazakii*, they found that some strains contained either a toxin or an antitoxin component but not both. Only 55 of the 63 strains tested possessed three of these genes (*fic*, *relB*, and *parDE*), pointing to possible nucleotide polymorphisms at these loci or to the absence of the genes. Additionally, the only strains that contained all 22 TA homologs were the *C. sakazakii* ST1.

Five strains of *C. sakazakii* were resistant *in vitro* to cephalothin, four to ampicillin, and two to ceftazidime, amoxicillin/clavulanic acid and nalidixic acid. In addition, one strain was resistant to four of the 10 antibiotics tested (40%) and two strains to two antibiotics (20%) (Table 4). Resistance of *C. sakazakii* to cephalothin, ceftazidime, and ampicillin has been evidenced in several previous studies, and a quasi-intrinsic resistance to cephalothin by *Cronobacter* spp. has been proposed (Kim et al., 2008; Molloy et al., 2009; Flores et al., 2011; Chon et al., 2012). Among clinical strains of *C. sakazakii*, Holý et al. (2019) did not find antibiotic resistant strains. In contrast, a more recent study in five *C. sakazakii* strains isolated from powdered milk distributed in Latin America found 100% to be resistant to cefotaxime and ampicillin, 60% to cefepime, 40% to amikacin, and 20% to cephalothin. One strain of *C. sakazakii* was resistant to six of the 12 antibiotics tested (54.5%), while another strain was resistant to five (50%) (Parra-Flores et al., 2020). These outcomes should be studied further due to the emergence of multidrug-resistant *C. sakazakii* strains, such as the one causing neonatal meningitis in China that was resistant to eight antibiotics (Zeng et al., 2018), which represents a clear health risk for infants.

**TABLE 4:** Antibiotic resistance profile of *Cronobacter sakazakii* strains isolated of PIF.

	**Antibiotics**									
**Strains**	**CAZ (30 μg)**	**CTX (30 μg)**	**AMOX + AC (20/10 μg)**	**CIP (5 μg)**	**CF (30 μg)**	**NAL (30 μg)**	**GE (10 μg)**	**TC (30 μg)**	**CL (30 μg)**	**AMP (10 μg)**
CH42	S	S	I	S	R	S	S	S	S	R
CH43	R	S	S	S	I	S	S	S	S	R
CH44	S	S	R	S	R	R	S	S	S	R
CH45	S	S	S	S	I	R	S	S	S	R
CH50	S	S	S	S	R	S	S	S	S	I
CH65	R	S	R	S	R	S	S	S	S	S
CH84	S	S	S	S	R	S	S	S	S	I

*R, resistant; I, intermediate; S, susceptible. CAZ, Ceftazidime; CTX, Cefotaxime; AMOX + AC, Amoxicillin + clavulanic acid; CIP, Ciprofloxacin; CF, Cephalothin; NAL, Nalidixic acid; GE, Gentamicin; TC, Tetracycline; CL, Chloramphenicol; AM, Ampicillin.*

Regarding the *in silico* presence of antibiotic resistance genes, all strains of *C. sakazakii* had the same efflux genes (*adeF*, *H-NS*, *msbA*, *marA*, *kpnF*, *kpnE*, *emrR*, *emrB*, *rsmA*, and *cRP*), one antibiotic inactivation gene (*ampH*), and four antibiotic target alteration genes (*pBP3*, *glpT*, *eF-Tu*, and *marR*), which confer antibiotic resistance to beta-lactams, fluoroquinolones, aminoglycosides, and phosphonates (Table 5). The *marA* gene, whose transcriptional function regulates multidrug efflux and modulates membrane permeability, was found in all isolates. Aly et al. (2019) found *msbA*, *emrR*, *H-NS*, *emrB*, *marA*, *CRP*, and *PBP3* to be associated with resistance to several antibiotics. Lepuschitz et al. (2019) found that out of 21 *C. sakazakii* isolates, 12 carried the efflux genes *emrB*, *msbA*, and *patA*; the antibiotic-efflux-modulating regulatory system genes *CRP*, *marA*, *emrR*, *marR*, and *H-NS*; the antibiotic target protection gene *msrB*; and the fosfomycin resistance determinant *glpT*. This aspect becomes particularly relevant in the context of increasing antibiotic resistance (Falagas et al., 2019) considering that fosfomycin is considered a useful antibiotic for patients with multidrug-resistant bacterial infections and since in our study the *glpT* gene was found in 100% of the isolates.

**TABLE 5:** Antibiotic-resistance genes identified by Comprehensive Antibiotic Resistance Database (CARD) of *C. sakazakii* strains.

**Best Hits Antibiotic Resistance Ontology (ARO)**	**Drug class**	**Resistance mechanism**	**CH42**	**CH43**	**CH44**	**CH45**	**CH50**	**CH65**	**CH84**
*MCR-9.1*	peptide antibiotic	antibiotic target alteration	+	+	+	+	−	+	+
*pBP3*	cephalosporin, cephamycin, penam	antibiotic target alteration	+	+	+	+	+	+	+
*glpT*	fosfomycin	antibiotic target alteration	+	+	+	+	+	+	+
*eF-Tu*	elfamycin antibiotic	antibiotic target alteration	+	+	+	+	+	+	+
*marR*	fluoroquinolone antibiotic; triclosan; rifamycin antibiotic; penam; phenicol antibiotic; glycylcycline; tetracycline antibiotic; cephalosporin	antibiotic target alteration	+	+	+	+	+	+	+
*adeF*	fluoroquinolone antibiotic, tetracycline antibiotic	antibiotic efflux	+	+	+	+	+	+	+
*H-NS*	macrolide antibiotic, fluoroquinolone antibiotic, cephalosporin, cephamycin, penam, tetracycline antibiotic	antibiotic efflux	+	+	+	+	+	+	+
*msbA*	nitroimidazole antibiotic	antibiotic efflux	+	+	+	+	+	+	+
*marA*	fluoroquinolone antibiotic, monobactam, carbapenem, cephalosporin, glycylcycline, cephamycin, penam, tetracycline antibiotic, rifamycin antibiotic, phenicol antibiotic, triclosan, penem	antibiotic efflux	+	+	+	+	+	+	+
*kpnF*	macrolide antibiotic, aminoglycoside antibiotic, cephalosporin, tetracycline antibiotic, peptide antibiotic, rifamycin antibiotic	antibiotic efflux	+	+	+	+	+	+	+
*kpnE*	macrolide antibiotic, aminoglycoside antibiotic, cephalosporin, tetracycline antibiotic, peptide antibiotic, rifamycin antibiotic	antibiotic efflux	+	+	+	+	+	+	+
*emrR*	fluoroquinolone antibiotic	antibiotic efflux	+	+	+	+	+	+	+
*emrB*	fluoroquinolone antibiotic	antibiotic efflux	+	+	+	+	+	+	+
*rsmA*	fluoroquinolone antibiotic, diaminopyrimidine antibiotic, phenicol antibiotic	antibiotic efflux	+	+	+	+	+	+	+
*cRP*	fluoroquinolone antibiotic; macrolide antibiotic; penam	antibiotic efflux	+	+	+	+	+	+	+
*kpnH*	macrolide antibiotic, fluoroquinolone antibiotic, aminoglycoside antibiotic, carbapenem, cephalosporin, penam, peptide antibiotic, penem	antibiotic efflux	−	−	−	−	−	−	−
*baeR*	aminoglycoside antibiotic, aminocoumarin antibiotic	antibiotic efflux	−	−	−	−	−	−	−
*ampH ampC-type beta-lactamase*	cephalosporin, penam	antibiotic inactivation	+	+	+	+	+	+	+
*fosA5*	fosfomycin	antibiotic inactivation	−	−	−	−	−	−	−

Antibiotic overuse in food environments and the presence of several antibiotic resistance operons (*marA*) can favor the development of resistance to different antibiotics in *Cronobacter* spp. (Kim et al., 2008; Chon et al., 2012; Holý et al., 2021). In addition, we found the *mcr-9.1* and *bla*_*CSA*_ genes, conferring resistance to colistin and cephalothin, respectively. The *mcr-9.1* gene is considered a plasmid-borne colistin resistance gene that can generate colistin resistance in various enteropathogens. These genes can silently circulate undetected unless induced by colistin (Carroll et al., 2019; Kieffer et al., 2019). The presence of mobile colistin-resistant (*mcr*) genes causes worldwide concern because colistin is considered the last resort for treating infections caused by multidrug-resistant *Enterobacteriaceae* (Borowiak et al., 2020). Müller et al. (2014) first described the β-lactamase class C resistance gene family *bla*_*CSA*_. Members of this family of β-lactamases are not inducible and are considered cephalosporinases. Jang et al. (2020) found class C *bla* resistance gene variants identified as *CSA-2* or *CSA-1*. On the other hand, Holý et al. (2021) found *bla*_*CSA*_ genes providing cephalothin resistance in all *C. sakazakii* strains isolated from powdered milk produced in the Czechia between 2010–2014.

All isolates studied here carried plasmids Col440I and Col (pHHAD28) and one isolate IncFII (pECLA), which are associated with antibiotic resistance genes (Ramsamy et al., 2020; Khezri et al., 2021). The presence of *terC*, from the plasmid-encoded tellurium resistance (*ter*) operon, is highly associated with infected patients compared to asymptomatic colonized patients. Furthermore, it is associated with pathogenesis of *Klebsiella pneumoniae* as a horizontally transferable factor that promotes robust intestinal colonization in the presence of the autochthonous microbiota (Vornhagen et al., 2020).

All *C. sakazakii* strains showed CRISPR arrays, and three strains had both I-E and I-F type arrays. CRISPR-Cas systems are related to the acquisition of horizontally acquired genetic material and have been recognized as an immunity system. These systems acquire information by means of viruses and plasmids. They consist of a guide RNA (gRNA) and a non-specific endonuclease associated with Cas-encoding genes. In the present study, when analyzing the genomes of the seven strains of *C. sakazakii*, we found that 100% (7/7) presented repeated sequences and spacers, forming arrays associated with CRISPR systems type I-F and I-E (Table 6). Regarding the CRISPR arrays, it was found that in system type I-E, there were five different consensus repeat sequences associated with it, of which the sequences GTGTTCCCCGCGCGAGCGGGGATAAACCG and CTGTTCCCCGCGCGAGCGGGGATAAACCG were the most frequent and were found in six of the seven strains in the study. In the case of the type I-F system, two different associated repeated sequences were found, of which the sequence GTTCACTGCCGTACAGGCAGCTTAGAAA was the most frequent and the sequence TTTCTAAGCTGCCTGTAC GGCAGTGAAC was characteristic of strains CH43 and CH84. Even though the sequences of each system may be the same, the numbers and lengths of repeated sequences and spacers differentiate them. With respect to the above, it was found that strains CH42, CH43, CH44, CH45, and CH84 presented the largest arrays, with up to a maximum of 30 repeat sequences and 29 spacers in the case of system I-E and 13 repeat sequences and 12 spacers in system I-E.

**TABLE 6:** Profiling of CRISPR-Cas loci among *C. sakazakii* strains.

**Strains**	**Identification**	**Operon estructure type**	**Number of CRISPRs arrays per strain**	**Maximun number of spacers per strains**	**Repeat consensus**	**Cas genes**
CH42	*Cronobacter sakazakii*	CAS-Type I-F CAS-Type I-E	13 27 30	12 26 29	GTTCACTGCCGTACAGGCAGCTTAGAAA CTGTTCCCCGCGCGAGCGGGGATAAACCG/GTGTTCCCCGCGCGAGCGGGGATAAACCG	*cas3, cse1, cse2, cse4, cas5e, cse3, cas1, cas2*
CH43	*Cronobacter sakazakii*	CAS-Type I-E	27 30 13	26 29 12	CTGTTCCCCGCGCGAGCGGGGATAAACCG GTGTTCCCCGCGCGAGCGGGGATAAACCG TTTCTAAGCTGCCTGTACGGCAGTGAAC	*cas3, cse1, cse2, cse4, cas5e, cse3, cas1, cas2*
CH44	*Cronobacter sakazakii*	CAS-Type I-E CAS-Type I-F	27 30 13	26 29 12	CTGTTCCCCGCGCGAGCGGGGATAAACCG GTGTTCCCCGCGCGAGCGGGGATAAACCG GTTCACTGCCGTACAGGCAGCTTAGAAA	*cas3, cse1, cse2, cse4, cas5e, cse3, cas1, Cas2*
CH45	*Cronobacter sakazakii*	CAS-Type I-F CAS-Type I-E	13 27 30	12 26 29	GTTCACTGCCGTACAGGCAGCTTAGAAA CTGTTCCCCGCGCGAGCGGGGATAAACCG GTGTTCCCCGCGCGAGCGGGGATAAACCG	*cas3, cse1, cse2, cse4, cas5e, cse3, cas1, cas2*
CH50	*Cronobacter sakazakii*	CAS-Type I-E	8 10	7 9	GTGTTCCCCGCGCGAGCGGGGATAAACCG GGTTTATCCCCGCTCGCGCGGGGAACAC	*cas3, cse1, cse2, cse4, cas5e, cse3, cas1, cas2*
CH65	*Cronobacter sakazakii*	CAS-Type I-E	13	12	GTTCACTGCCGTACAGGCAGCTTAGAAA	*cas3, cse1, cse2, cse4, cas5e, cse3, cas1, cas2*
CH84	*Cronobacter sakazakii*	CAS-Type I-E	13 27 30	12 26 29	TTTCTAAGCTGCCTGTACGGCAGTGAAC CTGTTCCCCGCGCGAGCGGGGATAAACCG GTGTTCCCCGCGCGAGCGGGGATAAACCG	*cas3, cse1, cse2, cse4, cas5e, cse3, cas1, cas2*

The relevance of studying the diversity of the CRISPR genes is that these systems can be used as a typing method of different microorganisms. Ogrodzki and Forsythe (2016), in analyzing 29 *C. sakazakii* ST1 genomes, found the same operon structure type I-E and three spacer arrays with conserved patterns. In addition, CRISPR spacer matrix profiles allow better intraspecies discrimination than MLST, so they have been proposed as a future tool for epidemiological studies of outbreaks by *Cronobacter* species (Makarova and Koonin, 2015). Although it was initially proposed that *C. sakazakii* had only one type of CRISPR system, Zeng et al. (2017) and Ogrodzki and Forsythe (2017) found that 94.5% of *C. sakazakii* strains had more than one CRISPR array, which were present in conserved areas of their genomes. The *cas1* and *cas2* genes are indispensable for the integration and processing of information acquired by the bacterium: In their absence, the system loses the ability to acquire information.

In our study, the use of WGS led to the reidentification of *C. sakazakii* and the determination of multiple virulence and antibiotic resistance genes in PIF and dairy products intended for consumption by infants. This situation should be analyzed in greater depth due to the growing international commercialization of powdered milk as a base product to manufacture other dairy products and byproducts. In Chile, legislation has just been passed regarding the obligation of producers of PIF and dairy products to disclose the origin of the powdered milk that is reused to prepare fluid milks and dairy by products. This requirement seeks to maintain an active record of information for authorities and supply visible information on the product’s retail label so consumers can make informed choices.

Considering that powdered milks are consumed by those who are susceptible to *Cronobacter* infection, the presence of virulence factors and antibiotic resistance in strains of *C. sakazakii* isolated from these products should be more closely monitored due to the direct relationship they have with the severity of the disease associated with this pathogen. Therefore, health authorities need to carry out more activities with preventive control measures for these foods along with campaigns to encourage the use of powdered milk rehydration water at 70°C, as indicated by the World Health Organization, which reports that this temperature has a proven effect of significantly decreasing the risk of *C. sakazakii* disease in already reconstituted milks (Parra-Flores et al., 2018b).

## Conclusion

*Cronobacter sakazakii* strains isolated from powdered infant formula and powdered milk showed diverse virulence factors as well resistance to beta-lactam antibiotics *in silico* and *in vitro*. These findings reinforce the governmental decision to recall all involved powdered and dairy formulas in Chile in 2017. Continued surveillance of these products is necessary due to the risk associated with product contamination by *C. sakazakii* and consumption by the immunologically vulnerable infant population.

## Data Availability Statement

The datasets presented in this study can be found in online repositories. The names of the repository/repositories and accession number(s) can be found below: https://pubmlst.org/organisms/cronobacter-spp/, 3195–3201.

## Author Contributions

JP-F, OH, SL, AC-C, MT, and GF conceived the experiments and prepared the manuscript. JP-F, AC-C, SL, and MT conducted the laboratory work. JP-F, FR, EM-S, AR-F, JX-C, WR, and SF drafted the manuscript. All authors reviewed and approved the final manuscript.

## Conflict of Interest

The authors declare that the research was conducted in the absence of any commercial or financial relationships that could be construed as a potential conflict of interest.

## Footnotes

1 http://pubmlst.org/Cronobacter

2 http://www.genomicepidemiology.org

3 https://crisprcas.i2bc.paris-saclay.fr

4 http://crispr.otago.ac.nz/CRISPRDetect/predict_crispr_array.html
