# Supplementary material for: Profiling the Virulence and Antibiotic Resistance Genes of Cronobacter sakazakii Strains Isolated From Powdered and Dairy Formulas by Whole-Genome Sequencing
Source: Front Microbiol. 2021 Jun 30;12:694922. doi: 10.3389/fmicb.2021.694922 (PMC8278472; doi:10.3389/fmicb.2021.694922)
Supplement: Supplementary file 1 [file Table_1.DOCX]

**Table supplementary 1**. Plasmids present in *C. sakazakii* strains isolated from PIF and PM

| **Strains** | **Plasmids** | **Position in contig.** | **Accession number** | **Function** |
| --- | --- | --- | --- | --- |
| CH42  CH43  CH44  CH45  CH50 | Col440l  Col440l  Col440l  Col(pHAD28)  Col440l  Col440l | 3663  236  1531  3856  3838  396 | CP023920.1  CP023920.1  CP023920.1  KU674895  CP023920.1  CP023920.1 | Antibiotic resistance |
| CH65 | Col440l | 474 | CP023920.1 |  |
